# Pilot study on developing a decision support tool for guiding re-administration of chemotherapeutic agent after a serious adverse drug reaction

**DOI:** 10.1186/1471-2407-11-319

**Published:** 2011-07-28

**Authors:** Pei Yi Loke, Lita Chew, Chun Wei Yap

**Affiliations:** 1 Department of Pharmacy, National University of Singapore, Singapore; 2 Department of Pharmacy, National Cancer Centre Singapore, Singapore

## Abstract

**Background:**

Currently, there are no standard guidelines for recommending re-administration of a chemotherapeutic drug to a patient after a serious adverse drug reaction (ADR) incident. The decision on whether to rechallenge the patient is based on the experience of the clinician and is highly subjective. Thus the aim of this study is to develop a decision support tool to assist clinicians in this decision making process.

**Methods:**

The inclusion criteria for patients in this study are: (1) had chemotherapy at National Cancer Centre Singapore between 2004 to 2009, (2) suffered from serious ADRs, and (3) were rechallenged. A total of 46 patients fulfilled the inclusion criteria. A genetic algorithm attribute selection method was used to identify clinical predictors for patients' rechallenge status. A Naïve Bayes model was then developed using 35 patients and externally validated using 11 patients.

**Results:**

Eight patient attributes (age, chemotherapeutic drug, albumin level, red blood cell level, platelet level, abnormal white blood cell level, abnormal alkaline phosphatase level and abnormal alanine aminotransferase level) were identified as clinical predictors for rechallenge status of patients. The Naïve Bayes model had an AUC of 0.767 and was found to be useful for assisting clinical decision making after clinicians had identified a group of patients for rechallenge. A platform independent version and an online version of the model is available to facilitate independent validation of the model.

**Conclusion:**

Due to the limited size of the validation set, a more extensive validation of the model is necessary before it can be adopted for routine clinical use. Once validated, the model can be used to assist clinicians in deciding whether to rechallenge patients by determining if their initial assessment of rechallenge status of patients is accurate.

## Background

The chemotherapeutic drug class was identified as the most common class for adverse drug reactions (ADR), accounting for 21.8% of 408 ADRs reported in an Indian hospital [1]. Patients on chemotherapy may experience serious ADRs which can be potentially fatal and may require costly interventions. 10.5% of 4075 women on chemotherapy had hospitalizations or emergency room visits for serious ADRs, resulting in an additional $1271 per person annually [2]. However, re-introduction of chemotherapeutic agents may be required due to the lack of alternative treatments. Currently, there are no standard guidelines for recommending re-administration of the chemotherapeutic drug to the patient after a serious ADR incident. Thus, management decisions are based purely on the experience of the clinicians and are highly subjective. Although patients with mild ADRs can usually be re-administered with the drug without much risks, there is still no consensus on whether patients with serious ADRs should be re-administered with the drug. This is because there are currently no methods which can accurately identify patients who will have negative rechallenge (ADRs do not re-occur upon re-administration of drug) or positive rechallenge (ADRs re-occur upon re-administration of drug). Thus, it will be useful to have a method that can predict patients' rechallenge status in order to improve patient safety and assist in chemotherapy choices.

Data mining is the use of sophisticated data analysis tools to discover patterns and relationships in large data set [3]. It can build computational models from data sets by learning from past experiences. Applications of data mining have been widely used in many diverse areas like business, medical research and pharmacovigilance. For example, Nordyke et al. proposes the use of Naïve Bayes in the automated diagnosis of thyroid dysfunction [4]. Hence, data mining methods could potentially be useful in analyzing an ADRs database and identifying important clinical predictors for patients' rechallenge status.

The aim of this study is to develop a clinical decision support tool, using data mining methods, to assist in determining the appropriateness of re-introducing a chemotherapeutic agent following the confirmatory association between the drug and the occurrence of a serious ADR in a patient. A Naïve Bayes method was used to analyze differences between the profiles of patients with negative rechallenge and those with positive rechallenge, and to develop a model to predict the patients' rechallenge status. Naïve Bayes was selected as it has been shown to produce relatively good classification performance and is straightforward in implementation [5]. A genetic algorithm (GA) attribute selection method was used to identify clinical predictors that could differentiate patients with negative challenge from those with positive rechallenge. GA can identify several combinations of clinical predictors that are equally good. This allows analysis of the results and selection of the most clinically relevant attributes as predictors.

## Methods

### Data collection

A total of 854 patients who were treated at the National Cancer Centre Singapore (NCCS) during the period 2004-2009 and experienced ADRs from chemotherapeutic agents were identified using spontaneous ADR reporting forms. Among these patients, 81 experienced serious ADR and 46 of them were rechallenged. These include 23 negative rechallenge cases and 23 positive rechallenge cases. Information about these 46 patients' demographics, relevant medical records and laboratory values were collected from the case notes and electronic medical systems.

### Preprocessing

The dataset was split into 3 sets: training, testing and validation. A total of 24 cases were randomly selected from the 35 cases that occurred between 2004 to 2008 to form the training set for developing the Naïve Bayes model. All the 35 cases were used as the testing set to test the performance of the model during the GA attribute selection process. The 11 cases that occurred during 2009 were used as an independent validation set to validate the model. These 11 cases were not used during the development of the model or during attribute selection.

### Patient's attributes

Patient's attributes that may have potential associations with rechallenge status and which were readily available were collected for the 46 patients. The attributes, given in Table 1, can be broadly categorized into the following groups: (1) patient demographics, (2) medical conditions, (3) medications usage, (4) laboratory parameters. Genetic variables were not included in the study as genetic testing is not commonly conducted in NCCS.

**Table 1:** Patient's attributes that were collected in this study

Attributes			
*Demographics*	- Age	- Gender	- Ethnicity
	- Weight		
*Medical Conditions*	- Drug allergy	- Cancer type	- Cancer malignancy
	- Comorbidities	- Symptoms of the ADRs	- Hospitalization for prior
	- ADRs affected organ systems	- Onset of ADRs	ADRs
*Medications*	- Number of cycles	- Chemotherapeutic drug	- Chemotherapeutic drug class
	- Number of doses	- Concurrent medications	
	- Dose reduction on rechallenge	- Rechallenge on same day	
*Laboratory parameters*	- White blood cell	- Red blood cell	- Eosinophil
	- Neutrophil	- Platelet	- Monocyte
	- Lymphocyte	- Serum creatinine	- Alkaline Phosphatase
	- Alanine aminotransferase	- Aspartate aminotransferase	- Basophil
			- Albumin

Discrete categories of attributes were derived from some of the continuous attributes. For example, age was expressed as a continuous variable and a discrete variable with 2 categories: elderly (65 years old and above) and non-elderly (less than 65 years old) [6]. The number of concurrent medications was also categorized into a binomial attribute to test for possible associations between polypharmacy and rechallenge status. The definition of "more than 5 concomitant drugs" for serious polypharmacy [7] was adopted in this study. In addition to the presence of comorbidities, the number of comorbidities for each patient was also calculated. These comorbidities include hypertension, diabetes, hyperlipidemia, psoriasis, gastroestrophageal reflux disease (GERD), asthma and other allergic disorders. Other additional attributes constructed include the class of chemotherapeutic agents, types and malignancy of cancer, and types of ADRs experienced.

Laboratory parameters used include full blood count (white blood cell, red blood cell, platelet, neutrophil, lymphocyte, monocyte, eosinophil and basophil levels), renal indices (serum creatinine) and liver indices (aspartate aminotransferase, alanine aminotransferase and alkaline phosphatase). These are routinely analyzed for all patients in NCCS and the laboratory panels were obtained from the most recent measurements before the reported ADR incident. Additional attributes representing the presence or absence of abnormal laboratory parameters were also constructed.

The total number of attributes used for data mining was 53. Detailed information on these 53 attributes is provided in Additional File 1: Appendices 1 to 3.

### Genetic Algorithm Attribute Selection

**Figure 1:**
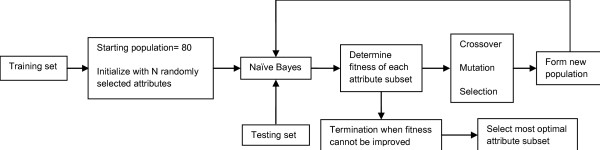
**Overview of the Genetic Algorithm attribute selection process**.

GA was used to rank and select attributes that are useful for predicting patients' rechallenge status. Figure 1 shows an overview of the GA attribute selection process. The process involved creating multiple 'generations' of attribute subsets. Each attribute subset contained between 1 to 53 attributes that were initially randomly chosen. During each 'generation', a Naïve Bayes model was developed using the training set for each of the 80 attribute subsets in the population. A fitness value was determined for each model by using the testing set to compute the area under the receiver operating characteristic curve (AUC). The roulette wheel method was used as the selection operator for reproduction and population replacement. Crossover and mutation rates of 95% and 5% were used respectively to construct new attribute subsets for the next 'generation'. This process was repeated until 100 'generations' was reached. The GA attribute selection process was repeated 5 times using different starting 'generation' of attribute subsets. The best attribute subset with the highest AUC from these 5 runs was chosen. The clinical predictors were then selected from this attribute subset by expert assessment of the Naïve Bayes distribution table and clinical significance of the predictors. The final Naïve Bayes model was then developed using the 35 cases that occurred between 2004-2008 and the selected clinical predictors.

### Naïve Bayes

Naïve Bayes is a simple probabilistic classification algorithm based on Bayes' theorem and has the assumption of conditional independence of the predictive attributes [8,9]. Given a set of patient attributes, F = {F_1_, F_2_... F_n_}, the posterior probability of a patient having negative rechallenge (NR) under the assumption of independence of the attributes can be computed as follows:

### Performance Evaluation

The performance of the model was measured using AUC, which is frequently used to evaluate prediction models in the biomedical informatics field [10,11]. In addition, the sensitivity and specificity of the model were calculated. Sensitivity refers to the proportion of patients with negative rechallenge who are predicted to have negative rechallenge. Specificity refers to the proportion of patients with positive rechallenge who are predicted to have positive rechallenge.

## Results

### Clinical Predictors

The set of clinical predictors identified by GA and refined by expert assessment is listed in Table 2. Patients' rechallenge status was found to be associated with age. The mean age for positive rechallenge cases were higher (55.55 ± 14.18) compared to negative rechallenge cases (47.71 ± 14.87). The type of chemotherapeutic agents administered was also found to be a significant clinical predictor, with patients who were given carboplatin, paclitaxel, docetaxel and cetuximab being more likely to have positive rechallenge. The mean albumin level for patients with positive rechallenge was 30.9 g/l, compared to 35.7 g/l for patients with negative rechallenge. Mean platelet and red blood cell (RBC) levels were found to be lower in patients who have positive rechallenge. Patients with abnormal levels of alanine aminotransferase (ALT), alkaline phosphatase (AP) and white blood cell (WBC) were more likely to have positive rechallenge.

**Table 2:** Descriptive statistics of clinical predictors in testing set (n = 35)

Numerical attributes	Negative rechallenge (Mean +/- SD)	Positive rechallenge (Mean +/- SD)
Age	47.7 +/- 14.9	55.4 +/- 14.2
Albumin level	35.7 +/- 4.6	30.9 +/- 5.6
RBC level	4.06 +/- 0.58	3.69 +/- 0.81
Platelet level	331.41 +/- 153.62	324.39 +/- 150.86
		

**Categorical and binominal attributes**	**Negative rechallenge (proportion)**	**Positive rechallenge (proportion)**

**Chemotherapeutic drug**		
Oxaliplatin	0.294	0.278
Carboplatin	0.059	0.278
Bleomycin	0.059	0.000
Rituximab	0.235	0.000
Paclitaxel	0.118	0.167
Docetaxel	0.059	0.111
Trastuzumab	0.118	0.056
Cetuximab	0.000	0.056
Gemcitabine	0.059	0.056
		
**Laboratory parameters**		
Abnormal WBC level*	0.118	0.444
Abnormal ALT level*	0.118	0.278
Abnormal AP level*	0.176	0.556

*Presence of abnormal level is allocated value of 1 while absence of abnormal level is allocated value of 0.

### Naïve Bayes model

The Naïve Bayes model developed using the 35 cases that occurred between 2004-2008 and the selected clinical predictors had an AUC of 0.767 for the validation set.

A platform independent version and an online version of the model (PaDEL-Rechallenge) is available at http://padel.nus.edu.sg/software/padelrechallenge. This will facilitate independent validation of the model by clinicians.

## Discussion

### Clinical predictors

It is important to note that the identified clinical predictors in this study were only found to be associated with rechallenge status. However, association does not imply causality. It is also essential to note that most of these were weak associations, but when considered together in the Naïve Bayes model, they were found to be useful for predicting patients' rechallenge status. Detailed discussion on the individual predictors can be found in Additional File 1: Appendix 4.

### Performance

Since there are no similar studies that develop models for predicting patients' rechallenge status, we will assess the performance of our model by making tentative comparisons with other models developed in other biomedical fields. A Naïve Bayes and Radial Basis Function method for predicting implantation potentials of IVF embryos reported superior performance with an AUC of 0.712 [12]. Another model that used artificial neural network model to differentiate between patients with and without prostate cancer had an AUC range of 0.77 to 0.81 [13]. Thus, our Naïve Bayes model with an AUC of 0.767 can be considered to have acceptable prediction performance.

It is important to note that the rechallenge status of those patients who were not rechallenged will never be known. Since our model was not trained using this group of patients, it is not justifiable to replace clinical judgement with our model for predicting the rechallenge status for all patients. Instead, a more suitable application for our model will be to assist in subsequent clinical decision making after clinicians had identified a group of patients who are likely to have negative rechallenge.

The 13 serious ADR cases that occurred in 2009 will be used to illustrate the potential usefulness of our model for this type of application. Initial clinical judgement identified 11 cases as potentially negative rechallenge cases. Out of these, 6 were negative rechallenge cases and 5 were positive rechallenge cases. Thus initial clinical judgement had a sensitivity of 100% and specificity of 0%. Our Naïve Bayes model can be used to improve the prediction accuracy of the clinicians by providing a score for each case. A threshold for the score can be set and patients with scores above or below this threshold will be predicted by the model as potential negative or positive rechallenge cases respectively. Clinicians can choose different thresholds for the score according to their treatment objectives for the patients. For example, a low threshold is more important for patients who are undergoing curative treatment so that they are not deprived of a useful drug treatment. This is a significant issue in chemotherapeutic treatment because there are limited choices of effective chemotherapeutic drugs available to the patients. Thus, the benefits from using the first line drugs usually outweigh the potential risks caused by any serious ADRs. The sensitivity and specificity of our model using 0.01 as the threshold is 100% and 20% respectively. Conversely, a high threshold would be more useful for patients undergoing palliative chemotherapeutic treatment as the key priority is to prevent them from experiencing unnecessary serious ADRs caused by rechallenge. A threshold of 0.8 would result in a sensitivity of 67% and specificity of 80% for our model.

### Limitations

Despite analyzing 6 years worth of data, the size of the dataset used to develop and validate our model is rather small. This is due to the limited number of ADR cases reported and collected in NCCS. Thus, this study is only a pilot study and there is a need for further validation of the accuracy and reproducibility of this model. A study is currently ongoing to validate the model using 2010 to 2014 data. In addition, a visual aid will be added to improve the interpretability of the results by clinicians. Additional discussion on other limitations of this study can be found in Additional File 1: Appendix 4.

## Conclusion

Compared to clinical judgement, the Naïve Bayes model developed in this study is able to guide rechallenge decisions more consistently and thus allows clinicians to make more confident decisions on whether to rechallenge a patient with the same drug after a prior serious ADR. The proposed use of the model is to assist in subsequent clinical decision making after the clinicians had identified a group of patients for rechallenge. Thus the model serves as a subsequent check to reinforce or discourage the initial decision for rechallenge. This would help to reduce serious ADRs and improve patient's treatment options.

## Competing interests

The authors declare that they have no competing interests.

## Authors' contributions

PYL carried out the acquisition of data, performed attribute selection and model development, analyzed and interpret the results, and drafted the manuscript. LC took part in the study design, advised on the data collection, and proposed critical revisions to the draft manuscript. CWY took part in the study design, advised on the attribute selection and model development, analyzed and interpret the results, and proposed critical revisions to the draft manuscript. All authors read and approved the final manuscript.

## Pre-publication history

The pre-publication history for this paper can be accessed here:

http://www.biomedcentral.com/1471-2407/11/319/prepub

## Supplementary Material

Additional file 1 **Appendices**. Contains Appendix 1: Detailed list of patient's attributes collected or derived in this study Appendix 2. Descriptive statistics for numerical attributes in testing and validation set Appendix 3. Descriptive statistics for nominal attributes in testing and validation set Appendix 4. Additional discussion Click here for file
