# Supplementary material for: Pilot study on developing a decision support tool for guiding re-administration of chemotherapeutic agent after a serious adverse drug reaction
Source: BMC Cancer. 2011 Jul 28;11:319. doi: 10.1186/1471-2407-11-319 (PMC3161037; doi:10.1186/1471-2407-11-319)
Supplement: Additional file 1 — Appendices. Contains Appendix 1: Detailed list of patient's attributes collected or derived in this study Appendix 2. Descriptive statistics for numerical attributes in testing and validation set Appendix 3. Descriptive statistics for nominal attributes in testing and validation set Appendix 4. Additional discussion [file 1471-2407-11-319-S1.DOC]

Appendix 1 - Detailed list of patient’s attributes collected or derived in this study

| **Attribute** | **Type** | **Comments** |
| --- | --- | --- |
| ETHNIC | DISCRETE | CHINESE, MALAY, INDIAN |
| CHINESE | DISCRETE |  |
| FEMALE | DISCRETE |  |
| AGE | CONTINUOUS |  |
| ELDERLY | DISCRETE | <65years old, ≥65 years old |
| WEIGHT | CONTINUOUS |  |
| DRUG ALLERGIES | DISCRETE | Presence of drug allergy |
| CHEMO DRUG | DISCRETE | Oxaliplatin, Paclitaxel, Rituximab, Carboplatin, Trastuzumab, Docetaxel, Gemcitabine, Cetuximab, Bleomycin |
| CHEMO DRUG TYPE | DISCRETE | Platinum, monoclonal antibodies, taxoids, others |
| DOSE | CONTINUOUS | Number of dose of chemotherapeutic drug received during rechallenge |
| CYCLE | CONTINUOUS | Number of cycle of chemotherapeutic drug received during rechallenge |
| INDICATION | DISCRETE | Lung, breast, colorectal, germ cell cancer, lymphoma and other cancers |
| METASTASIS | DISCRETE | Presence of cancer metastasis |
| ADRpul | DISCRETE | ADRs associated with pulmonary, upper respiratory tract (e.g wheezing, breathing difficulty, chest tightness) |
| ADRcv | DISCRETE | ADRs associated with cardiovascular system (e.g. hypotension, hypertension, tachycardia, arrhythmias) |
| ADRskin | DISCRETE | ADRs associated with skin reactions (e.g rigours, chills, flushing, rash) |
| ADRgi | DISCRETE | ADRs associated with gastrointestinal system (e.g nausea, dysphagia) |
| FEVER | DISCRETE | Onset of fever symptoms during ADR |
| NO OF CONCURRENT CHEMO DRUGS | CONTINUOUS | Number of concurrent chemotherapeutic drugs |
| NO. OF CONCURRENT DRUGS | CONTINUOUS | Number of concurrent drugs (excluding chemotherapeutic drugs) |
| POLYPHARMACY | DISCRETE | More than 5 concurrent drugs taken |
| HOSPITALISATION | DISCRETE | Hospitalization for previous ADR |
| ACUTE | DISCRETE | ADRs within 24 hours of administration |
| RECHALLENGE ON SAME DAY | DISCRETE | Rechallenge done on the same day as ADR onset |
| DOSE REDUCTION | DISCRETE | Dose reduction for rechallenge of same chemotherapeutic drug |
| ALBUMIN | CONTINUOUS | Albumin level |
| WBC | CONTINUOUS | White blood cell count |
| PLATELET | CONTINUOUS | Platelet count |
| RBC | CONTINUOUS | Red blood cell count |
| EOSINOPHIL | CONTINUOUS | Eosinophil level |
| NEUTROPHIL | CONTINUOUS | Neutrophil level |
| LYMPHOCYTES | CONTINUOUS | Lymphocyte level |
| MONOCYTES | CONTINUOUS | Monocyte level |
| BASOPHILS | CONTINUOUS | Basophil level |
| SCr | CONTINUOUS | Serum Creatinine level |
| AP | CONTINUOUS | Alkaline Phosphatase level |
| ALT | CONTINUOUS | Alanine Aminotransferase level |
| AST | CONTINUOUS | Aspartate Aminotransferase level |
| COMORBIDITES | DISCRETE | Presence of any comorbidities |
| NO. OF COMORBDITIES | CONTINUOUS | Number of comorbidities |
| Abalbumin* | DISCRETE | Normal range: 37-51g/l |
| Abplatelet* | DISCRETE | Normal range: 140-440 X 10^9^/l |
| Abeosinophil* | DISCRETE | Normal range: 0-6% |
| Abneutrophil* | DISCRETE | Normal range: 40-75% |
| Ablymphocyte* | DISCRETE | Normal range: 15-41% |
| Abmonocyte* | DISCRETE | Normal range: 2-10% |
| AbSCr* | DISCRETE | Normal range: 40-85umol/l |
| Abalt* | DISCRETE | Normal range: 7-36u/l |
| Abast* | DISCRETE | Normal range: 15-33U/l |
| Abrbc* | DISCRETE | Normal range: 4.5-6.3 X 10^12^/l |
| Abwbc* | DISCRETE | Normal range: 4.1-10 X 10^9^/l |
| Abap* | DISCRETE | Normal range: 32-103 u/l |
| Abbasophil* | DISCRETE | Normal range: 0-1% |

*Ab indicates abnormal levels. For example, Abalbumin means that the patient had abnormal albumin levels. Normal ranges of laboratory parameters are based on guidelines from the NCCS Laboratory Department.

Appendix 2 - Descriptive statistics for numerical attributes in testing and validation set

| **Attributes** | **Mean +/- SD [Range]** | |
| --- | --- | --- |
|  | **Testing Set (n=35)** | **Validation Set (n=11)** |
| AGE | 51.7 +/- 14.8 [22.0 ; 75.0 ] | 56.1 +/- 14.0 [34.0 ; 72.0] |
| WEIGHT | 59.9 +/- 15.3 [37.2 ; 105.5] | 59.1 +/- 13.3 [36.6 ; 78.8] |
| DOSE | 3.6 +/- 2.5 [1.0 ; 15.0] | 4.1 +/- 2.5 [1.0 ; 10.0] |
| CYCLE | 2.8 +/- 1.6 [1.0 ; 7.0] | 2.8 +/- 1.4 [1.0 ; 6.0] |
| NO OF CONCURRENT CHEMO DRUGS | 1.1 +/- 0.8 [0.0 ; 3.0] | 0.8 +/- 0.9 [0.0 ; 3.0] |
| NO. OF CONCURRENT DRUGS | 4.2 +/- 2.1 [1.0 ; 8.0] | 4.8 +/- 3.3 [1.0 ; 12.0] |
| ALBUMIN | 33.26 +/- 5.59 [21.00 ; 40.00] | 33.36 +/- 4.01 [26.00 ; 39.00] |
| WBC | 7.68 +/- 4.73 [2.90 ; 24.45] | 6.20 +/- 2.09 [3.30 ; 9.14] |
| PLATELET | 327.80 +/- 149.99 [91.00 ; 801.00] | 253.91 +/- 82.59 [64.00 ; 348.00] |
| RBC | 3.87 +/- 0.724 [2.52 ; 5.13] | 3.71 +/- 0.54 [2.78 ; 4.67] |
| EOSINOPHIL | 3.71 +/- 9.82 [0.00 ; 59.00] | 1.56 +/- 1.17 [0.10 ; 3.80] |
| NEUTROPHIL | 63.70 +/- 18.06 [15.00 ; 93.70] | 58.17 +/- 17.19 [14.00 ; 72.10] |
| LYMPHOCYTES | 22.59 +/- 13.83 [3.00 ; 55.40] | 30.19 +/- 18.21 [12.70 ; 78.00] |
| MONOCYTES | 10.96 +/- 14.36 [0.40 ; 89.00] | 9.32 +/- 3.56 [4.00 ; 14.20] |
| BASOPHILS | 0.46 +/- 0.36 [0.00 ; 1.90] | 0.51 +/- 0.23 [0.20 ; 1.00] |
| SCr | 68.46 +/- 20.96 [26.00 ; 117.00] | 68.00 +/- 17.53 [42.00 ; 91.00] |
| AP | 141.91 +/- 150.57 [17.00 ; 636.00] | 90.27 +/- 65.59 [33.00 ; 277.00] |
| ALT | 93.03 +/- 390.21 [7.00 ; 2,333.00] | 28.09 +/- 27.50 [11.00 ; 107.00] |
| AST | 242.31 +/- 1,223.59 [15.00 ; 7,273.00] | 27.09 +/- 16.03 [14.00 ; 63.00] |
| NO. OF COMORBDITIES | 0.9 +/- 1.2 [0.0 ; 4.0] | 1.8 +/- 1.3 [0.0 ; 4.0] |

Appendix 3 - Descriptive statistics for nominal attributes in testing and validation set

| **Attributes** | **Total Number** | |
| --- | --- | --- |
|  | **Testing Set (n=35)** | **Validation Set (n=11)** |
| ELDERLY | 8 | 5 |
|  |  |  |
| ETHNIC |  |  |
| Chinese | 32 | 10 |
| Malay | 2 | 1 |
| Indian | 1 | 0 |
|  |  |  |
| FEMALE | 21 | 7 |
| DRUG ALLERGIES | 8 | 2 |
|  |  |  |
| CHEMO DRUGS |  |  |
| Oxaliplatin | 10 | 2 |
| Carboplatin | 6 | 0 |
| Paclitaxel | 5 | 4 |
| Docetaxel | 3 | 0 |
| Rituximab | 4 | 2 |
| Cetuximab | 1 | 1 |
| Trastuzumab | 3 | 1 |
| Gemcitabine | 2 | 0 |
| Bleomycin | 1 | 1 |
|  |  |  |
| CHEMO DRUG TYPE |  |  |
| Platinum | 16 | 2 |
| Taxoid | 8 | 4 |
| Monoclonal antibodies | 8 | 4 |
| Others | 3 | 1 |
|  |  |  |
| INDICATION |  |  |
| Lung cancer | 3 | 1 |
| Breast cancer | 9 | 2 |
| Colorectal cancer | 11 | 2 |
| Germ cell cancer | 4 | 2 |
| Lymphoma | 5 | 2 |
| Others | 3 | 2 |
|  |  |  |
| METASTASIS | 28 | 6 |
| ADRpul | 18 | 7 |
| ADRcv | 18 | 5 |
| ADRskin | 26 | 8 |
| ADRgi | 7 | 2 |
| FEVER | 2 | 1 |
| POLYPHARMACY | 11 | 11 |
| HOSPITALISATION | 14 | 3 |
| ACUTE | 34 | 11 |
| RECHALLENGE ON SAME DAY | 8 | 2 |
| DOSE REDUCTION | 8 | 1 |
| COMORBIDITES | 18 | 9 |
| Abalbumin | 21 | 8 |
| Abplatelet | 10 | 1 |
| Abeosinophil | 3 | 0 |
| Abneutrophil | 12 | 1 |
| Ablymphocyte | 15 | 3 |
| Abmonocyte | 16 | 6 |
| AbSCr | 10 | 3 |
| Abalt | 7 | 2 |
| Abast | 14 | 4 |
| Abrbc | 27 | 10 |
| Abwbc | 10 | 3 |
| Abap | 13 | 1 |
| Abbasophil | 2 | 0 |

**Appendix 4 – Additional discussion**

**Clinical predictors**

Age was identified as a clinical predictor in this study. This is consistent with evidence from various clinical studies [1, 2]. Older patients are at greater risk for ADRs associated with drug interactions and toxicity due to their altered pharmacokinetic and pharmacodynamic status. They are also more likely to have some form of abnormalities in their bodies’ drug distribution, metabolism, excretion or cellular response to the drugs.

The type of chemotherapeutic drug used by the patients was also identified as a clinical predictor. Patients who encountered prior serious ADRs to docetaxel, paclitaxel, carboplatin and cetuximab were more likely to have positive rechallenge. For carboplatin, this is in concordance with previous studies which found that at least 50% of patients would develop repeat hypersensitivity reactions when rechallenged with platinum drugs [3, 4].

In this study, low albumin level was found to be associated with greater risk of positive rechallenge. This is similar to the findings from a few studies [5, 6]. There are two possible reasons for the importance of albumin level for predicting patients’ rechallenge status. Firstly, the amount of free drug for highly protein-bound drugs like paclitaxel and docetaxel are affected by the albumin level [7, 8]. The effect could be further potentiated by drug displacement due to the use of multiple drugs, leading to dose-dependent toxicity [9]. Secondly, the albumin level could serve as an indicator of a person’s nutritional status and patients with poorer nutritional status have been associated with higher incidences of ADRs [10].

In this study, red blood cell count, platelet count and presence of abnormal white blood cell count were selected as clinical predictors for patients’ rechallenge status. Both low platelet count and red blood cell count had been identified as possible clinical predictors of ADRs in a few epidemiological studies [11-13]. However, the underlying mechanisms of these two factors in causing serious ADRs in patients have yet to be elucidated. For white blood cells, they can release cytokine and activate other cells of the innate immune system, resulting in an amplification of the inflammatory response. Hence, abnormally high WBC levels may potentially lead to greater immune-mediated ADR response by the body towards the chemotherapeutic agents.

Alanine aminotransferase and alkaline phosphatase may have been selected as clinical predictors because abnormal levels of these two enzymes are indicative of some form of liver disease or damage. The CYP450 enzymes, which are found predominantly in the liver, are mainly responsible for the metabolic elimination of chemotherapeutic drugs like paclitaxel and docetaxel. Thus, impaired hepatic clearance may result in increased risk of ADR events due to the accumulation of active parent compounds or drug metabolites. These reactive molecules may also covalently bind to larger macromolecules like host proteins and trigger an immune response.

**Other limitations**

A limitation of the current study is that some confounding variables were not considered during the development of the Naïve Bayes model. The decision on whether to rechallenge was highly subjective and varies among clinicians. In addition, pre-rechallenge measures to reduce the risk of serious ADRs such as desensitization, use of pre-medications like corticosteroids or antihistamines, and prolonging drug infusion were not standardized for all patients. Thus, these factors may not be similar in the negative rechallenge and positive rechallenge groups. Hence, our identification of clinical predictors may be confounded by these differences. Another potentially confounding variable was the use of traditional Chinese medicine (TCM), which may be a significant clinical predictor for patients’ rechallenge status. There is high prevalence of TCM use among patients in NCCS [14] and this could result in interactions with the chemotherapeutic drugs, thus causing the occurrence of ADRs. However, due to the lack of patient records on the use of TCM, this potentially useful predictor cannot be included for developing the model. Nonetheless, it is easy to rebuild the model to include these clinically relevant attributes when more information is available in the future.

The current model is only suitable for guiding rechallenge decisions in patients undergoing chemotherapy since it was developed using such information. However, the model is expected to work well for other therapeutic areas after slight modifications and retraining with relevant information from these therapeutic areas since most of the clinical predictors are patient’s attributes that are not unique to chemotherapy.

**References**

1. Jose J, Rao PG: **Pattern of adverse drug reactions notified by spontaneous reporting in an Indian tertiary care teaching hospital**. *Pharmacol Res* 2006, **54**(3):226-233.

2. Beijer HJ, de Blaey CJ: **Hospitalisations caused by adverse drug reactions (ADR): a meta-analysis of observational studies**. *Pharm World Sci* 2002, **24**(2):46-54.

3. Lafay-Cousin L, Sung L, Carret AS, Hukin J, Wilson B, Johnston DL, Zelcer S, Silva M, Odame I, Mpofu C *et al*: **Carboplatin hypersensitivity reaction in pediatric patients with low-grade glioma: a Canadian Pediatric Brain Tumor Consortium experience**. *Cancer* 2008, **112**(4):892-899.

4. Siu SW, Chan RT, Au GK: **Hypersensitivity reactions to oxaliplatin: experience in a single institute**. *Ann Oncol* 2006, **17**(2):259-261.

5. GonzalezMartin G, Yanez L, Valenzuela E: **A prospective study of adverse drug reactions among hospitalized elders**. *Rev Medica Chile* 1997, **125**(10):1129-1136.

6. Bowman L, Carlstedt BC, Hancock EF, Black CD: **Adverse drug reaction (ADR) occurrence and evaluation in elderly inpatients**. *Pharmacoepidemiol Drug Saf* 1996, **5**(1):9-18.

7. Hayes MJ, Langman MJ, Short AH: **Changes in drug metabolism with increasing age: 1. warfarin binding and plasma proteins**. *Br J Clin Pharmacol* 1975, **2**(1):69-72.

8. Hayes MJ, Langman MJ, Short AH: **Changes in drug metabolism with increasing age: 2. phenytoin clearance and protein binding**. *Br J Clin Pharmacol* 1975, **2**(1):73-79.

9. Wallace S, Whiting B: **Factors affecting drug binding in plasma of elderly patients**. *Br J Clin Pharmacol* 1976, **3**(2):327-330.

10. Caamano F, Pedone C, Zuccala G, Carbonin P: **Socio-demographic factors related to the prevalence of adverse drug reaction at hospital admission in an elderly population**. *Arch Gerontol Geriatr* 2005, **40**(1):45-52.

11. Zopf Y, Rabe C, Neubert A, Hahn EG, Dormann H: **Risk factors associated with adverse drug reactions following hospital admission: a prospective analysis of 907 patients in two German university hospitals**. *Drug Saf* 2008, **31**(9):789-798.

12. Bates DW, Miller EB, Cullen DJ, Burdick L, Williams L, Laird N, Petersen LA, Small SD, Sweitzer BJ, Vander Vliet M *et al*: **Patient risk factors for adverse drug events in hospitalized patients. ADE Prevention Study Group**. *Arch Intern Med* 1999, **159**(21):2553-2560.

13. Vakil BJ, Kulkarni RD, Chabria NL, Chadha DR, Deshpande VA: **Intense surveillance of adverse drug reactions. An analysis of 338 patients**. *J Clin Pharmacol* 1975, **15**(5-6):435-441.

14. Shih V, Chiang JY, Chan A: **Complementary and alternative medicine (CAM) usage in Singaporean adult cancer patients**. *Ann Oncol* 2009, **20**(4):752-757.
